# Systematic Review of Direct Hospital Costs Associated with Aneurysmal Subarachnoid Hemorrhage Management

**DOI:** 10.1007/s12028-025-02439-2

**Published:** 2026-01-15

**Authors:** Friso P. Mulder, Jeroen T. J. M. van Dijck, Samuel A. Corper, Rick J. G. Vreeburg, Wouter A. Moojen

**Affiliations:** Department of Neurosurgery, Leiden University Medical Center, Haaglanden Medical Center and Haga Teaching Hospital, University Neurosurgical Center Holland, Leiden and The Hague, the Netherlands

**Keywords:** Aneurysmal subarachnoid hemorrhage, Endovascular coiling, Health economics, In-hospital costs, Surgical clipping, Systematic review

## Abstract

**Background:**

Aneurysmal subarachnoid hemorrhage (aSAH) is a severe condition associated with significant morbidity, mortality, and disability. In-hospital care of aSAH is complex and resource intensive, posing substantial financial challenges to health care systems. To support efficient resource allocation, health policy decision-making, and care optimization, this systematic review aimed to assess and synthesize literature on direct in-hospital costs of aSAH management.

**Methods:**

A comprehensive search was performed in July 2025 across PubMed, Medline, Web of Science, Cochrane Library, Emcare, Embase (Ovid), and PsycINFO to identify studies reporting direct in-hospital costs related to aSAH. Reporting completeness and risk of bias were assessed using the Consolidated Health Economic Evaluation Reporting Standards 2022 and the Joanna Briggs Institute checklists. Reported costs were narratively synthesized and converted to 2024 US dollars using the CCEMG-EPPI-Centre Cost Converter. In addition, a random-effects meta-analysis was conducted to compare in-hospital costs between surgical clipping and endovascular coiling.

**Results:**

The database search identified 1,591 articles, of which 30 were included. The average reporting completeness was 74% (range 46–91%) and methodological quality 76% (range 41–100%). Reported in-hospital costs ranged from $11,884 to $459,579 (median $68,711), being the highest in North America, followed by Europe and Asia. Costs as a percentage of gross domestic product per capita ranged from 35 to 639%. Key cost drivers included length of stay, clinical severity, and complications. The meta-analysis found no significant cost difference between clipping and coiling (mean difference $3,057, 95% confidence interval –$11,597 to $17,710).

**Conclusions:**

In-hospital costs for aSAH management are substantial and vary widely due to differences in health care systems, study methodology, and clinical practices. The quality of economic evaluations remains inconsistent, underscoring the need for more standardized and transparent methodologies. As global health care spending increases, high-quality economic evidence is essential for equitable and sustainable care.

**Supplementary Information:**

The online version contains supplementary material available at 10.1007/s12028-025-02439-2.

## Introduction

Aneurysmal subarachnoid hemorrhage (aSAH) is a serious, life-threatening condition, with an average global incidence of 7.9 per 100,000 persons [1]. It is associated with significant morbidity, mortality, and long-term disability, placing a substantial socioeconomic burden on both patients and health care systems [2–4]. Advances in neurocritical care, imaging, and microsurgical and endovascular interventions have improved survival rates and reduced morbidity [5, 6]. However, these advances are accompanied by considerable resource requirements and health care costs [4].

With the health care expenditures rising worldwide, the financial implications of aSAH management threaten the sustainability and affordability of health care systems [7, 8]. Therefore, there is an increasing need for cost analyses to guide policymakers and health care professionals in efficient allocation of limited resources, health policy decision-making, and the optimization of care pathways [9, 10].

Although several studies have examined costs or charges associated with aSAH management, there is wide variation in findings of economic evaluations due to differences in health care systems across countries, study methodologies, patient populations, and treatment protocols [11]. Moreover, the quality of economic evaluations studies differs considerably because of the lack of standardization and transparency in reporting [12, 13]. This underscores the need for a comprehensive and standardized overview of the direct hospital costs related to aSAH management, which is lacking in the current literature.

Therefore, the objective of this systematic review was to provide a systematic evaluation and quality assessment of the existing literature on direct in-hospital costs associated with the management of aSAH.

## Methods

This systematic review was conducted in accordance with the Preferred Reporting Items for Systematic Reviews and Meta-Analyses guidelines [14]. The study protocol was registered in the PROSPERO International Prospective Register of Systematic Reviews under registration number CRD420251043502.

### Eligibility Criteria

This systematic review included studies published from 2013 onward that reported in-hospital costs or charges for adult patients (≥ 18 years) with a confirmed diagnosis of aSAH. Direct costs had to be separable from any other types of costs, such as postdischarge care, rehabilitation, and indirect costs (e.g., productivity loss, informal care). Studies with a cohort size of at most ten or with no full text available in English were excluded. Furthermore, reviews, meta-analyses, case reports, commentaries, and editorials were also excluded. There were no restrictions on types of medical and surgical interventions for aSAH (e.g., clipping, coiling, drain placement, medication).

### Literature Search

A final systematic literature search was performed with assistance of an experienced librarian on July 1, 2025. The following electronic databases have been searched: PubMed, Medline, Web of Science, Cochrane Library, Emcare, Embase Ovid, and PsycINFO. Moreover, the reference lists of all included articles have been searched for additional relevant articles. Relevant information about the search strategy can be found in Supplementary File 1.

### Article Selection and Data Collection

After removal of duplicates, two reviewers independently performed the primary screening of the articles based on the title and abstracts and selected all potentially eligible studies. Full-text versions of the articles were retrieved and reviewed for eligibility according to the inclusion and exclusion criteria. The same two reviewers independently extracted the data from the selected articles using a predefined, standardized extraction form. No automation tools were used in the data collection process. Data extracted from the articles included study characteristics (e.g., year of publication, country, study design, and sample size), cost-related outcomes (e.g., total in-hospital costs, length of stay [LOS], details on cost calculation, perspective, currency, year), and study population (age, aSAH severity, complications). Disagreements during screening and data extraction were resolved through discussion. If a consensus could not be reached, a third reviewer was consulted.

### Risk of Bias Assessment

Risk of bias of the included studies was assessed using the Consolidated Health Economic Evaluation Reporting Standards (CHEERS) 2022 statement and the Joanna Briggs Institute (JBI) checklist for economic evaluations [12, 15]. The CHEERS statement, developed to improve the reporting quality of health economic evaluations, was applied to assess the completeness and transparency of reporting. The JBI checklist was used to evaluate the methodological quality and internal validity. As CHEERS is a reporting guideline rather than a validated quality appraisal tool, it was used alongside the JBI checklist to ensure a comprehensive and standardized assessment of both methodological quality and reporting completeness. This combined approach was particularly important given the well-recognized heterogeneity in the quality of health economic evaluations.

The CHEERS statement consists of 28 items, and the JBI checklist consists of 11 items. All items were scored according to a predefined scoring manual that included the following options: yes (2), suboptimal (1), no (0), or not applicable, with equal weight assigned to each item. Final scores were calculated as a percentage of the maximum achievable score per study, excluding all nonapplicable items. Two reviewers independently performed the assessments, and any discrepancies were resolved through discussion or consultations with a third reviewer. Although CHEERS was not originally developed as a quantitative scoring instrument, it was adapted in this review to evaluate adherence to reporting standards and to facilitate comparison of reporting completeness across studies, consistent with previous systematic reviews.

### Effect Measures

Given the anticipated heterogeneity in health care systems, costing methodologies, patient populations, and treatment protocols, the primary synthesis of the data was narrative. The mean or median costs per patient from admission to discharge were descriptively summarized. When provided, measures of variability (e.g., standard deviation or interquartile range) were also reported. All costs were converted to 2024 US dollars (USD) using the CCEMG-EPPI-Centre Cost Converter, which uses gross domestic product (GDP) deflator index values and purchasing power parities conversion rates provided by the International Monetary Fund [16]. Costs from Lebanon were adjusted to 2022 USD, because of missing GDP deflator and purchasing power parities data for 2024. For between-study comparisons, hospital costs were expressed as a percentage of the country’s GDP per capita, using GDP values for the study year converted to 2024 USD. If the currency year was not reported, the final year of patient inclusion was used instead. For studies that reported costs per subgroup but not an overall cost, the weighted average was used to calculate the overall value for graphical presentation. Figures were designed with R version 4.5.0 and GraphPad Prism version 10.5.0.

### Synthesis Methods

In addition to the narrative synthesis, a meta-analysis was performed to assess differences in total in-hospital costs between neurosurgical clipping and endovascular coiling. Studies were eligible if they reported mean cost estimates for both procedures with sufficient statistical data to allow calculation of a between-group mean difference. Studies reporting hospital charges instead of actual costs were excluded. Both common-effect and a random-effects meta-analyses (restricted maximum likelihood estimator) were calculated, with the random-effects model specified as the primary analysis. The pooled-effect measure was the mean difference in costs (calculated as clipping minus coiling) with 95% confidence intervals (CIs). Statistical heterogeneity was assessed using the *I*^2^ statistic and *τ*^2^. Sensitivity analyses were performed to assess the robustness of the findings by repeating the meta-analysis with alternative study inclusion scenarios, such as excluding lower-quality studies and statistical outliers. A formal assessment of reporting bias (funnel plot and Egger’s test) was not feasible, because of the limited number of comparable studies that evaluated costs of clipping versus coiling [17].

### Certainty Assessment

The certainty of evidence for the pooled analysis of the cost difference between clipping and coiling was assessed using the Grading of Recommendations Assessment, Development and Evaluation (GRADE) approach [18]. This approach assesses several domains that influence confidence in the results, such as risk of bias, inconsistency across studies, indirectness, imprecision, and potential publication bias. Based on these domains, the certainty of the evidence of each outcome is classified into high, moderate, low, or very low. Descriptive outcomes (in-hospital costs, costs as a percentage of GDP per capita, and LOS) were not assessed using GRADE. These are context-dependent absolute values rather than effect estimates, and current Cochrane guidance does not recommend applying GRADE to such outcomes [19].

## Results

### Study Selection

**Fig. 1:**
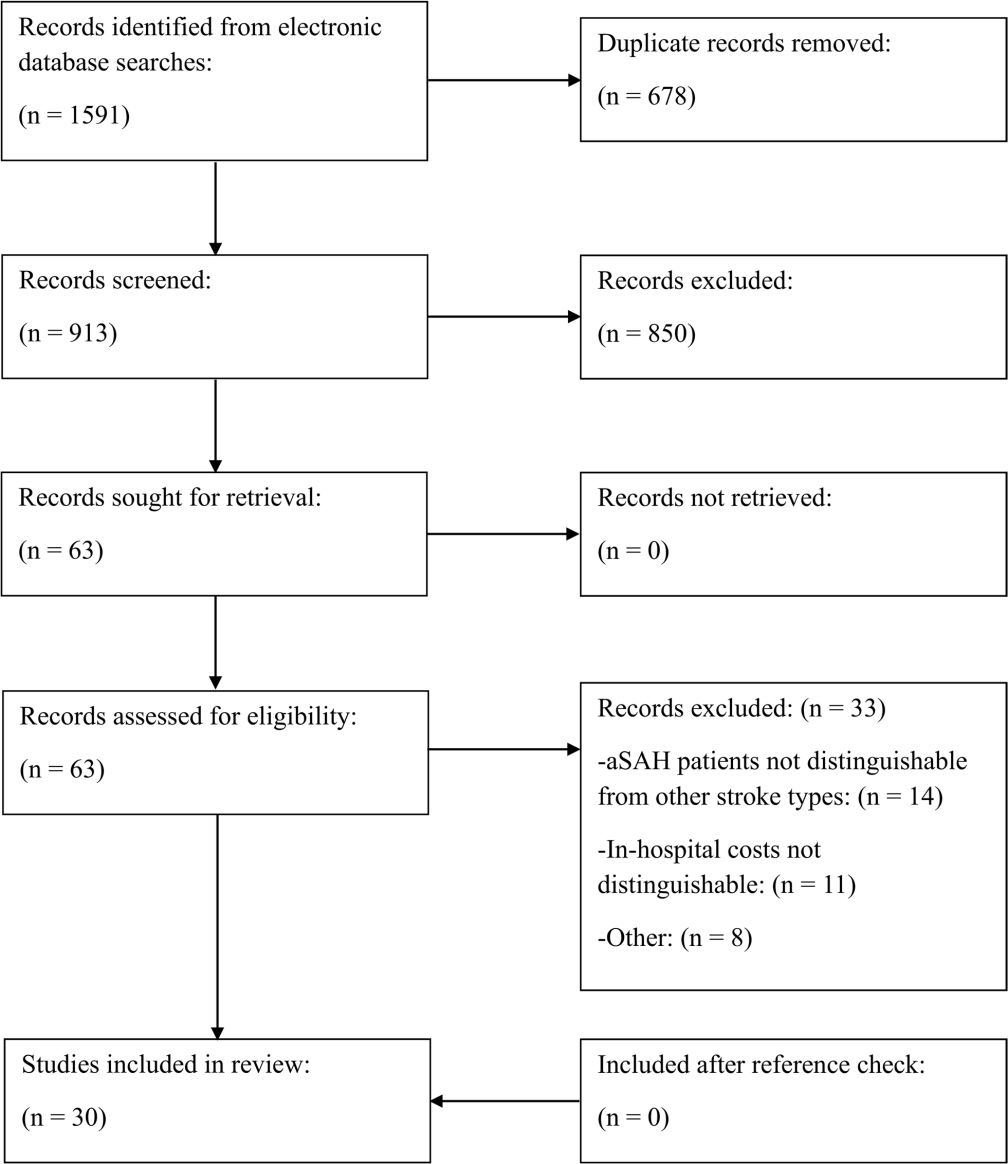
Flow diagram of the article selection process

A total of 1591 records were identified through searches across multiple databases. After the removal of 678 duplicates, 913 records remained for title and abstract screening. Of these, 63 full-text articles were assessed for eligibility. The most common reasons for exclusion were the inability to distinguish patients with aSAH from those with other stroke types (*n* = 14) and the inability to isolate in-hospital costs from other cost categories such as indirect or rehabilitation costs (*n* = 11). Screening of reference lists from included studies did not yield any additional eligible articles. Ultimately, 30 studies met the inclusion criteria and were included in the systematic review (Fig. 1) [20–49].

### Study Characteristics

Among the 30 included studies, 25 were retrospective cohort studies [21–23, 25–41, 43, 45–48], one was a retrospective temporal trend analysis [44], and one was a retrospective cross-sectional study [49] (Table 1). The remaining three were prospective cohort studies [20, 24, 42]. The majority of studies (*n* = 13) used a national database [22, 23, 25, 27, 31, 32, 37, 38, 41, 44, 46, 48, 49], whereas 12 were single-center studies [21, 26, 28–30, 33, 34, 36, 40, 42, 43, 45], and 5 were multicenter studies [20, 24, 35, 39, 47]. The sample size ranged from 13 to 127,155 patients, with a median of 327 patients [33, 42, 44].

**Table 1:** Overview of study characteristics and results

Study*	Currency (Year)		Sample Size	In-hospital Costs (2024 USD)		% of GDP**	Length of stay (days)	Source and calculation of costs	Study objective	Study type
Abdo^20^ 2018 2015–2016 Lebanon	USD (2015)	Total:	16	$394,231 ***	($271,509)^a^	1693% ***	31.1 (28.3)^a^	Billing department (8 hospitals) Bottom-up approach (unit cost x resource use)	Estimate stroke-related hospital costs and key cost-driving factors in Beirut hospitals	Observational, prospective, multicentre, cost-of-illness study
Abecassis^21^ 2019 2005–2017 USA	USD (NP)	**Overall** **Clipping:** **Coiling:**	88 33 55	$109,415 $161,870 $120,933	($66,150)^a^ ($68,854)^a^ ($51,328)^a^	145% 214% 160%	15.6 (13.1)^a^ 25.0 (13.8)^a^ 19.0 (10.2)^a^	Total actual costs from billing department (single centre)	Assess cost-effectiveness of coiling versus clipping for basilar tip aneurysms	Retrospective, single-centre, cohort study
Bekelis^22^ 2015 2005–2010 USA	USD (2010)	**Coiling:**	4311	$122,034	($119,327-$124,742)^b^	179%	15 (14)^c^	National Inpatient Sample database Charges converted to costs using average cost to charge ratio for each included hospital	Develop and validate a predictive model for hospitalization costs for cerebral aneurysm coiling	Retrospective cohort study with predictive modelling
Bekelis^23^ 2016 2005–2010 USA	USD (2010)	**Clipping:**	3293	$130,702	($127,224-$134,180)^b^	192%	20.21 (15.97)^a^	National Inpatient Sample database Charges converted to costs using average cost to charge ratio for each included hospital	Develop and validate a predictive model for hospitalization costs for cerebral aneurysm clipping	Retrospective cohort study with predictive modelling
Calciolari^24^ 2013 2005–2007 Italy	EUR (2012)	**H&H 1:** **H&H 2:** **H&H 3:** **H&H 4:** **H&H 5:**	17 49 21 13 7	$37,312 $40,050 $57,370 $54,089 $113,342	(NP)^a^ (NP)^a^ (NP)^a^ (NP)^a^ (NP)^a^	72% 77% 111% 104% 218%	17.02 (10–18)^d^ 21.22 (14–24)^d^ 25.57 (14–32)^d^ 19.18 (10–18)^d^ 38.14 (34–49)^d^	Resource consumption: questionnaire to14 hospitals Unit costs: hospital accounting department and regional office tariffs Bottom-up approach (unit cost x resource use)	Measure direct healthcare costs of intracranial aneurysm management	Observational, prospective, multicentre, cohort study
Chang^25^ 2016 2010–2014 South Korea	KRW (NP)	**Clipping:** **Coiling:**	7293 6954	$20,016 $24,026	($8987)^a^ ($11,279)^a^	40% 48%	15.0 (8.3)^a^ 8.6 (7.4)^a^	Hospital bills from Health Insurance Review & Assessment Service	Assess nation-wide cost-effectiveness of coiling versus clipping for Intracranial Aneurysms in Korea in patients aged > 30 years	Retrospective cohort study
Chen^26^ 2019 2010–2018 China	USD (NP)	**Total:**	32	$34,519	($20,083)^a^	174%	28 (12–42)^c^	No information provided on cost calculation, outcomes were obtained from electronic medical records (single centre)	Evaluate timing and outcomes in patients with haemorrhagic stroke who received tracheostomy	Retrospective, single-centre, cohort study
Deutsch^27^ 2018 2013–2014 USA	USD (NP)	**Clipping:** **Coiling:**	6555 15,350	**$**459,579 $448,803	($16,548)^a^ ($10,235)^a^	639% 624%	19.0 (33.2)^a^ 17.8 (32.2)^a^	Total hospital charges from the NIS database	Evaluate effects of clipping and coiling on risk-adjusted mortality and in-hospital complications for SAH	Retrospective cohort study
Fernando^28^ 2018 2011–2014 Canada	CAD (2017)	**Total:**	176	$95,943	($53,235)^a^	118%	27.5 (20.2)^a^	Case-costing system of the Ottawa Hospital Data Warehouse, which links total expenses to the hospital to the patient chart. (single centre)	Determine the overall outcomes and costs of ICU patients admitted with spontaneous intracranial haemorrhage	Retrospective, single-centre, cohort study
Koester^29^ 2022 2014–2019 USA	USD (NP)	**No DCI:** **DCI:**	247 144	$103,474 $134,605	($46,618)^a^ ($64,879)^a^	131% 171%	18 (8)^a^ 21 (11)^a^	Total costs from the Post-Barrow Ruptured Aneurysm Trial, which reflect actual expenses incurred by healthcare providers (single centre)	Analyse total hospital costs of DCI in aSAH	Retrospective, single-centre, cohort study
Labib^30^ 2022 2014–2019 USA	USD (NP)	**Clipping:** **Coiling:**	234 150	$113,462 $124,876	($54,696)^a^ ($53,519)^a^	144% 159%	20.1 (9.0)^a^ 18.7 (8.7)^a^	Total true costs from Strategic Support Services department of Dignity Health Arizona (single centre)	Compare total healthcare cost of endovascular versus microsurgical treatment of aSAH using a propensity-adjusted analysis and identify significant cost drivers	Retrospective, single-centre, cohort study
Lee^31^ 2013 1997–2002 Taiwan	TWD (NP)	**Total:**	78	$17,111	($13,203-$21,019)^b^	47%	17.6 (19.9)^a^	Stroke-related claims to the National Health Insurance	Examine readmissions, mortality and medical cost during the first year after acute stroke and explore predictive factors at the healthcare system level	Retrospective cohort study
Modi^32^ 2019 2002–2014 USA	USD (2017)	**Total:** **Clipping:** **Coiling:**	22,831 10,731 11,644	$103,299 $100,986 $102,929	($68,833)^a^ ($647)^e^ ($634)^e^	137% 134% 136%	16 (11–23)^c^ NP NP	National Inpatient Sample database Charges converted to costs using average cost to charge ratio for each included hospital	Estimate hospitalization costs of aSAH in the United States using a nationally representative administrative database and identify factors associated with higher costs	Retrospective cohort study
Monsivais^33^ 2019 2013–2015 USA	USD (2015)	**Clipping:** **Coiling:**	60 209	$95,593 $110,231	(NP)^a^ (NP)^a^	130% 150%	15 (10)^a^ 16 (10)^a^	Resource usage database and accounting database (single centre) Bottom-up approach (unit cost x resource use)	Compare resource usage and cost associated with each treatment modality in aSAH	Retrospective, single-centre, cohort study
Murata^34^ 2017 2008–2012 Japan	USD (NP)	**Total:**	27	$72,461	($67,960-$100,378)^c^	149%	53 (44–74)^c^	Medical records and inpatient claims (single centre) Fixed daily rate based on diagnosis and procedures performed	Investigate healthcare resource utilization and changes in functional status during consecutive inpatient stroke care	Retrospective, single-centre, cohort study
Ng^35^ 2015 2006–2012 Singapore	SGD (2012)	**Total:**	100	$36,352	($56,012)^a^	35%	39.3 (44.1)^a^	National Healthcare Group Bottom-up approach (unit cost x resource use) with costs calculated using before-subsidy charges	Identify total direct medical cost of the four main stroke types in Singapore	Retrospective, multicentre, cohort study
Ojha^36^ 2020 2006–2016 Australia	AUD (NP)	**Total:** **Alive:** **Deceased:**	139 50 89	$37,132 $99,846 $17,411	($8794-$86,433)^c^ ($78 438-$124,808)^c^ ($5863-$41,218)^c^	61% 163% 28%	8.8 (1.8–34.9)^c^ 38.3 (26.7–50.0)^c^ 4.1 (1.3–8.2)^c^	Hospital financial costs based on data obtained from medical records (single centre)	Describe survival rates, treatments provided, hospital discharge destination, number of organ donors and hospital financial costs in WFNS grade V aSAH patients	Retrospective, single-centre, cohort study
Raj^37^ 2018 2003–2013 Finland	EUR (2013)	**Total:**	1875	$36,100	($34,703-$37,498)^b^	63%	10 (6–16)^c^	Total costs from billing departments of hospitals in Finnish Intensive Care Consortium (FICC)	Describe differences in total one-year healthcare costs and one-year mortality and treatment cost-effectiveness following ICU admission after neurocritical disease	Retrospective cohort study
Ramos^38^ 2019 2002–2011 USA	USD (2017)	**Clipping:** **Coiling:**	18,318 11,931	$74,967 $77,527	(NP)^b^ (NP)^b^	99% 103%	16 (10–23)^c^ 15 (10–23)^c^	National Inpatient Sample database Charges converted to costs using average cost to charge ratio for each included hospital	Evaluate the effect of the safety-net burden on short-term inpatient outcomes, hospital costs, and quality metrics tied to reimbursement penalties for ruptured cerebral aneurysms	Retrospective cohort study
Rha^39^ 2013 2006 South Korea	KRW (2007)	**Total:**	125	$29,887	($26,091-$33,683)^b^	72%	32.8 (NP)^a^	Direct medical costs based on health insurance claims from 8 hospital electronic databases	Assess costs incurred for stroke treatment and care in Korea and identify factors related to the cost	Retrospective, multicentre, incidence-based, cohort study
Ridwan^40^ 2021 2007–2009 Germany	EUR (2015)	**Total:** **Clipping:** **Coiling:**	101 46 55	$43,753 $50,805 $37,855	(NP)^a^ (NP)^a^ (NP)^a^	67% 78% 58%	23.9 (NP)^a^ 27.5 (NP)^a^ 20.9 (NP)^a^	Costs/reimbursements based on DRG fixed case rates sourced from accounting office with detailed data on procedures and materials used (single centre)	Identify cost-driving factors for acute in-hospital treatment of aSAH	Retrospective, single-centre, cohort study
Rumalla^41^ 2018 2013 USA	USD (NP)	**Total:**	12,777	$80,591	($2458)^a^	114%	16 (0.3)^a^	Nationwide Readmissions Database Charges converted to costs using average cost to charge ratio for each included hospital	Analyse readmission rates and outcomes in patients with aSAH	Retrospective cohort study
Safanelli^42^ 2019 2016–2017 Brazil	USD (2016)	**Total:**	13	$11,884	($2982)^a^	99%	14 (6)^a^	Joinville Stroke Registry Bottom-up approach (unit cost x resource use)	Measure public in-hospital costs for ischemic stroke with and without cerebral reperfusion in Brazil	Prospective, prevalence-based, single-centre, cohort study
Seule^43^ 2020 2007–2010 Switzerland	EUR (2009)	**Total:** **WFNS 1–3:** **WFNS 4–5:**	150 108 42	$74,193 $61,180 $107,654	($67,005-$81,380)^b^ ($55,129-$67,231)^b^ ($91,083-$124,226)^b^	92% 76% 133%	17 (15–29)^c^ 16 (14.5–21)^c^ 27 (18–40)^c^	Hospital prospective aSAH database (single centre) Length of stay multiplied by average daily costs derived from Swiss Federal Statistical Office	Investigate outcome, return to work and costs after aSAH	Retrospective, single-centre, cohort study from a prospective database
Stepanova^44^ 2013 2005–2009 USA	USD (2009)	**2005:** **2006:** **2007:** **2008:** **2009:**	23,652 27,746 24,108 26,336 25,313	$56,855 $60,905 $61,219 $62,721 $60,012	($2919)^a^ ($3819)^a^ ($3980)^a^ ($3769)^a^ ($3229)^a^	85% 91% 92% 94% 90%	11.39 (0.38)^a^ 12.28 (0.51)^a^ 11.61 (0.46)^a^ 11.79 (0.47)^a^ 11.61 (0.42)^a^	National Inpatient Sample Costs estimated from charges using hospital-specific cost-to-charge ratios with national estimates reweighted to account for hospitals with missing cost-to-charge ratios	Evaluate changes in the in-hospital mortality and resource utilization for individuals admitted to the US hospitals with the principal diagnosis of stroke	Retrospective temporal trend study
Timmers^45^ 2021 2017–2019 Belgium	EUR (NP)	**WFNS 1–2:** **WFNS 3–5:**	44 25	$39,380 $84,113	($28,302-$44,930)^c^ ($38,307-$92,966)^c^	61% 131%	9 (2–13)^b^ 16 (3–63)^b^	Hospital data warehouse system (single centre) from healthcare payer perspective	Assess outcome and cost effectiveness in endovascular treatment of low and high grade aSAH and identify major cost drivers	Retrospective, single-centre, cohort study
Tong^46^ 2016 2003–2012 USA	USD (2012)	**Age 18–34:** **Age 35–44:** **Age 45–54:** **Age 55–64:** **Age 65–74:** **Age 75–84:** **Age > 85:**	3411 5282 11,408 11,848 7386 6039 3163	$69,480 $74,323 $75,517 $71,525 $63,039 $42,181 $29,467	(NP)^a^ (NP)^a^ (NP)^a^ (NP)^a^ (NP)^a^ (NP)^a^ (NP)^a^	100% 106% 108% 102% 90% 60% 42%	11.7 (0.5)^a^ 12.3 (0.4)^a^ 13.1 (0.3)^a^ 12.5 (0.3)^a^ 11.1 (0.3)^a^ 8.7 (0.4)^a^ 5.6 (0.3)^a^	National Inpatient Sample database Charges converted to costs using average cost to charge ratio for each included hospital	Determine the trends in hospitalizations caused by acute stroke by age groups	Retrospective cohort study
Xu^47^ 2024 2019–2022 China	CNY (2022)	**Total**	417	$26,648	(NP)^a^	109%	17.6 (18.4)^a^	Hospital information systems from primary, secondary and tertiary hospitals in Shenzhen	Estimate stroke-related hospitalization costs in Shenzhen, China	Retrospective cohort study
Yoon^48^ 2019 2002–2003; 2013–2015 USA	USD (2013)	**Clipping:** **Coiling:**	13,087 10,237	$124,080 $115,919	($3101)^e^ ($3158)^e^	175% 164%	20 (NP)^a^ 18 (NP)^a^	National Inpatient Sample database and Vizient Clinical Database Charges converted to costs using average cost to charge ratio for each included hospital	Analyse longitudinal trends in utilization and cost for clipping and coiling of ruptured aneurysms	Retrospective cohort study
Zhang^49^ 2019 2006–2013 China	CNY (2013)	**Total:**	1736	$21,228	($26,108)^a^	145%	25.2 (34.4)^a^	Urban Employee-based Basic Medical Insurance and Urban Resident-based Basic Medical Insurance claims databases based on payments to providers	Examine hospitalization costs for five stroke types and investigate factors associated with hospitalization costs	Retrospective, cross-sectional study

*Name first author^reference^; year of publication; patient inclusion period; study country

**The percentage of GDP per capita was calculated by converting the reported costs from the article to 2024 USD and dividing them by each country’s GDP per capita for the corresponding year, which was then also adjusted to 2024 USD

***Due to missing GDP deflator and PPP data for 2024, costs from Lebanon were adjusted to 2022 USD. The high costs and percentage of GDP are a result of the country’s severe economic crisis and hyperinflation

^a^Mean (Standard Deviation)

^b^Mean (95% Confidence Interval)

^c^Median (Interquartile Range)

^d^Mean (Interquartile Range)

^e^Mean (Standard Error)

*aSAH* Aneurysmal Subarachnoid Haemorrhage, *AUD* Australian Dollar, *CAD* Canadian Dollar, *CNY* Chinese Yuan, *DCI* Delayed Cerebral Ischemia, *DRG* Diagnosis-Related Group, *EUR* Euro, *GDP* Gross Domestic Product, *H&H* Hunt and Hess Grade, *ICU* Intensive Care Unit, *KRW* South Korean Won, *NIS* National Inpatient Sample, *NP* Not Provided, *SGD* Singapore Dollar, *TWD* Taiwan Dollar, *USD* United States Dollar, *WFNS* World Federation of Neurosurgical Societies

Most studies (*n* = 21) were conducted from a health care provider perspective [20–24, 27–30, 32, 33, 35, 36, 38, 39, 41, 42, 44, 46–48], whereas 7 studies adopted a payer perspective [25, 26, 31, 34, 40, 45, 49], and 2 adopted a societal perspective [37, 43]. Furthermore, 14 studies were conducted in North America [21–23, 26–30, 32, 33, 38, 41, 44, 46, 48], followed by 8 in Asia [25, 26, 31, 34, 35, 39, 47, 49], 5 in Europe [24, 37, 40, 43, 45], and 1 each in Australia [36], Brazil [42], and Lebanon [20]. In total, 25 studies were conducted in high-income countries.

### Study Quality and Risk of Bias

**Fig. 2:**
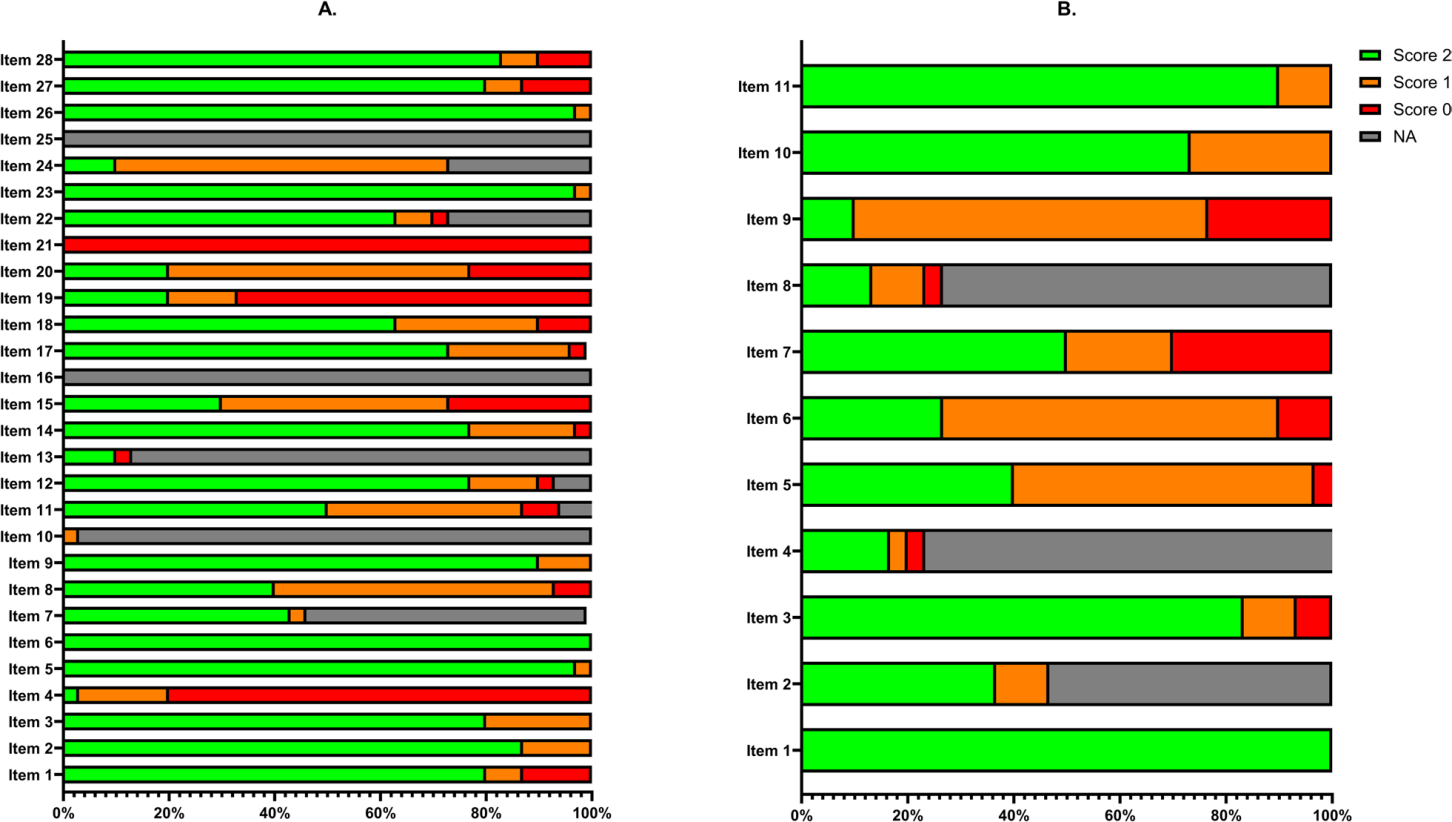
Quality assessment of included articles. Included articles were scored using **A** CHEERS checklist and **B** JBI checklist. Scores are categorized as follows: Score 2 (green) indicates the item was fully reported or met the criteria (optimal); Score 1 (orange) indicates partial reporting or suboptimal performance; Score 0 (red) indicates the item was not reported or did not meet the criteria; NA (gray) indicates the item was not applicable to the study. Details on the items and the scores for the individual studies can be found in Supplementary File 2.

Reporting completeness according to the CHEERS 2022 checklist ranged from 46 to 91% (mean 74%) (Supplementary File 2). Methodological quality according to the JBI checklist ranged from 41 to 100% (mean 76%). In total, there were 5 studies (17%) that scored 80% or above on both CHEERS and JBI [22–24, 37, 49]. Several checklist items were frequently underreported or not methodologically sound (Fig. 2). In particular, cost valuation and cost calculation were often not adequately addressed. A reference year for the costs was missing in 37% of the studies, and only 5 studies (17%) used the preferred bottom-up approach (unit costs × resource use) to calculate the costs. Equity considerations, such as characterizing distributional effects and engagement with patients and other stakeholders (CHEERS items 19 and 21), were rarely reported. Most studies performed well in reporting objectives, study population, setting, key findings, limitations, and generalizability.

### In-Hospital Costs

**Fig. 3:**
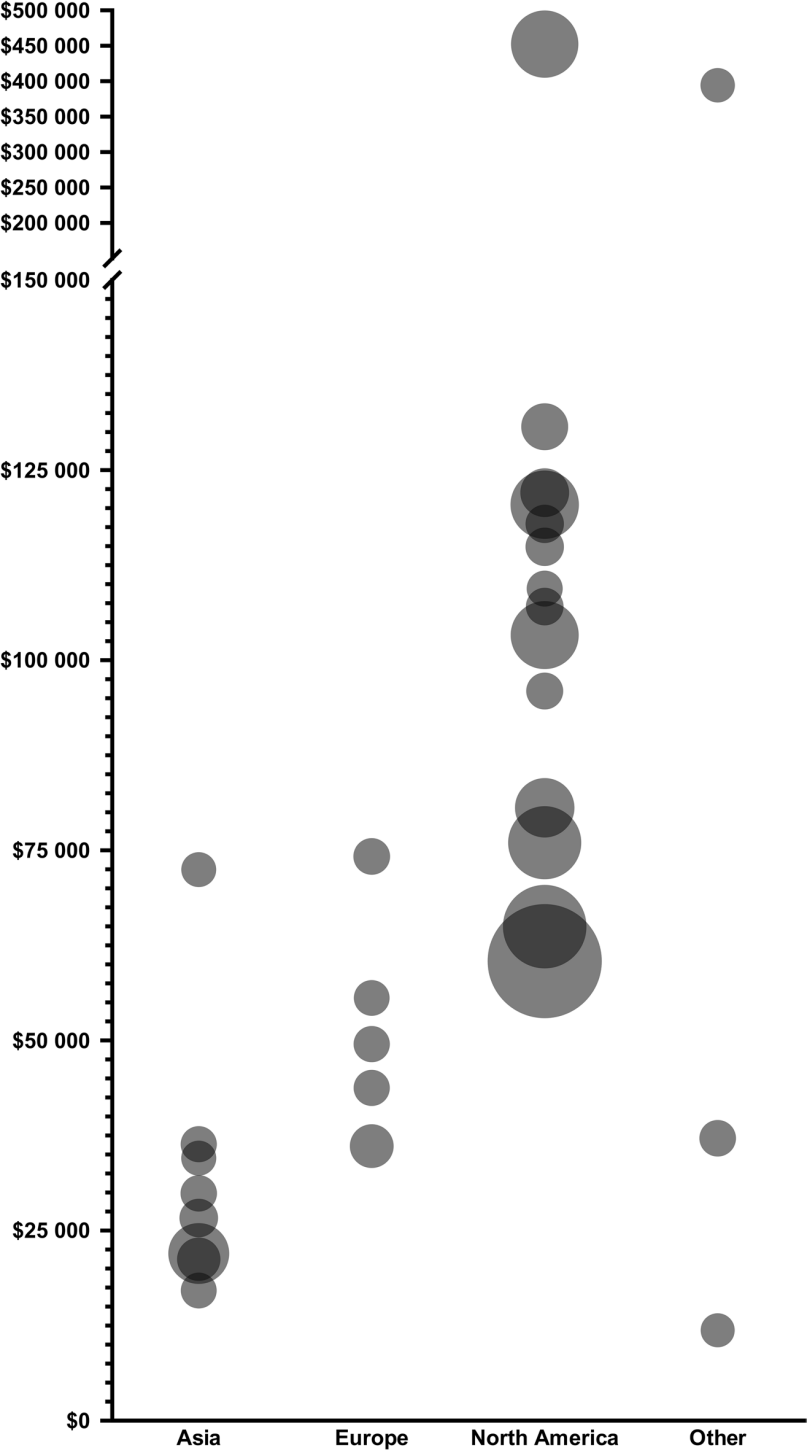
In-hospital costs per region. In-hospital costs per patient across regions in 2024 USD. Each circle represents a study, with circle size indicating the sample size

**Fig. 4:**
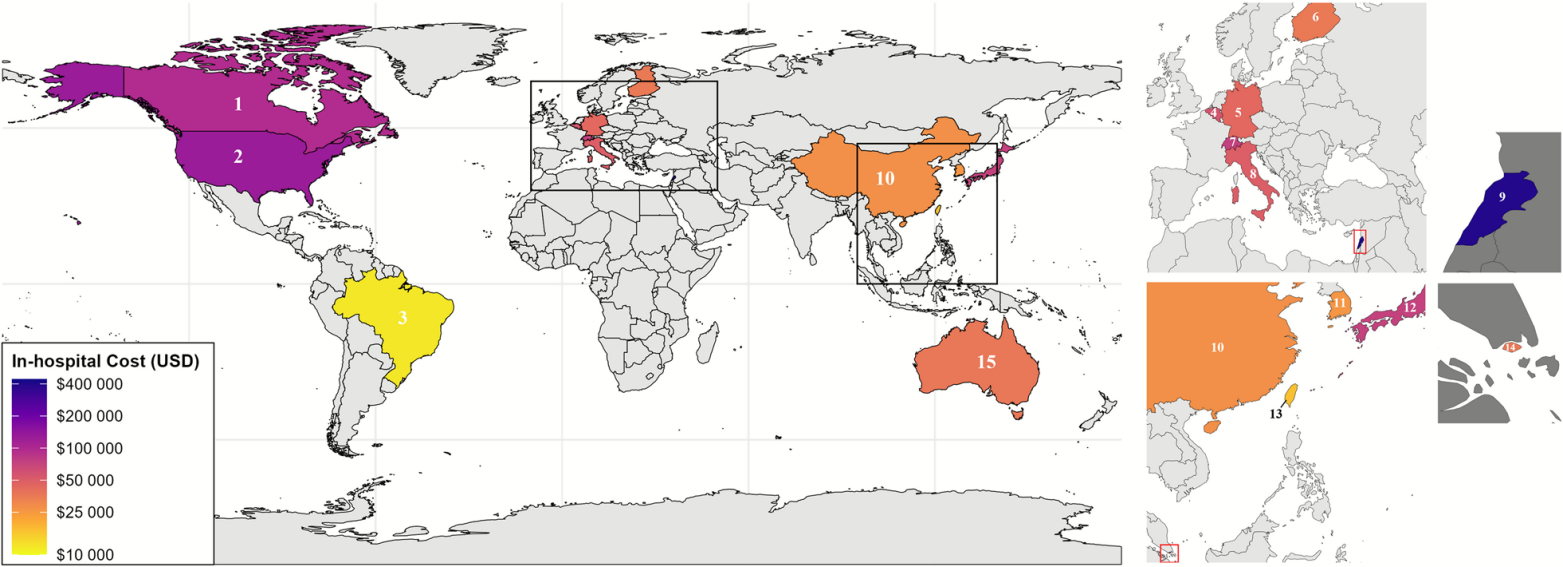
In-hospital cost per country. World map showing the mean in-hospital costs per country for aneurysmal subarachnoid hemorrhage (aSAH) in 2024 USD. Costs were log-transformed, with darker colors representing higher mean in-hospital costs (see legend). Countries are labeled numerically: (1) Canada, (2) United States, (3) Brazil, (4) Belgium, (5) Germany, (6) Finland, (7) Switzerland, (8) Italy, (9) Lebanon, (10) China, (11) South Korea, (12) Japan, (13) Taiwan, (14) Singapore, (15) Australia

**Fig. 5:**
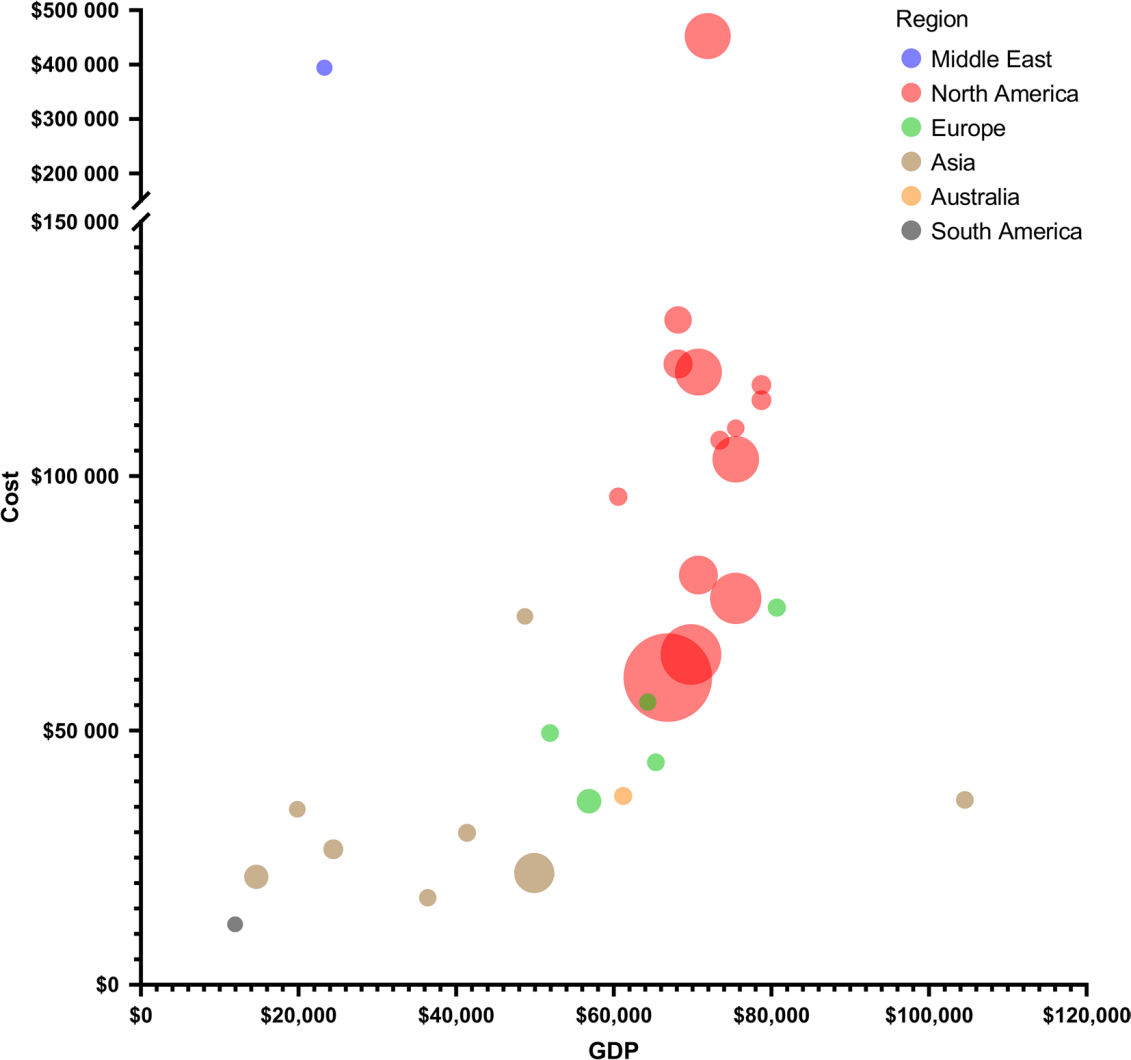
In-hospital costs versus GDP per Capita. Circle size reflects study sample size and the colors represent regions. For each study the GDP per capita was sourced from the International Monetary Fund based on the corresponding year and country, then adjusted using the CCEMG–EPPI-Centre Cost Converter. GDP per capita and in-hospital costs were converted to 2024 USD. *GDP* Gross Domestic Product

Reported in-hospital costs ranged from $11,884 to $459,579, with a median of $68,711 (Table 1). Studies conducted in North America reported the highest average costs at $121,902 (median $105,175, range $29,467–$459,579), followed by Europe (mean $51,829, median $49,515, range $36,100–$113,342) and Asia $32,523 (median $28,268, range $17,111–$72,461) (Figs. 3 and 4). The lowest mean costs ($11,884) were reported in Brazil [42]. One study from Lebanon reported a cost of $21,257 (2015 USD), which converted to $394,231 (2022 USD) because of the country’s severe economic crisis and hyperinflation [20]. As a percentage of national GDP per capita, North America had the highest median percentage (146%, range 42%–639%), followed by Asia (91%, range 35%–174%) and Europe (86%, range 58%–218%) (Table 1, Fig. 5). In addition to costs, in-hospital mortality was reported in 18 studies [20, 24, 26–29, 31, 32, 36, 38, 40–46, 48]. Mortality ranged from 11.9–31.8% in North America and 14.8–23.1% in Europe. In Asia, mortality showed a broader range, from 3.1–47.4%. The highest rate was reported in Brazil (53%).

Several studies reported a detailed breakdown of the in-hospital costs. Admission costs generally accounted for the biggest proportion ranging from 17–60% of the total costs. In line with this, LOS was consistently reported as a major cost driver. The median LOS for all studies was 17.6 days (range 8.8–53 days) (Table 1). Additionally, other substantial cost components included surgery (6–51%), imaging (2–12%), medication (1–18%), and laboratory services (1–14%) [20, 28, 31, 33, 36, 39, 42, 45, 49]. In-hospital rehabilitation costs were also reported in several studies, ranging from 0.6% to 36% of the total in-hospital costs [20, 30, 31, 34, 37, 39, 43].

Several studies identified factors associated with higher in-hospital costs. Clinical severity at admission significantly impacted the costs, with two studies reporting 1.76 to 2.14 times higher costs for patients with World Federation of Neurological Societies grades III–V [43, 45]. Complications were key cost drivers. In particular, delayed cerebral ischemia (DCI) was associated with a 25% to 30% increase in costs [21, 29, 45]. Similarly, hydrocephalus, seizures, and infectious complications (e.g., respiratory infections, meningitis) significantly increased costs [22, 23, 31, 32]. Furthermore, patients aged between 45 and 65 years generally incurred the highest costs, whereas patients aged more than 80 years incurred the lowest [32, 46, 49]. Looking at temporal trends, one study found no significant cost increase between 2005 and 2009 [44], whereas others reported increases of 7% to 42% over periods from 2002 to 2015 [25, 32, 46, 48].

**Fig. 6:**
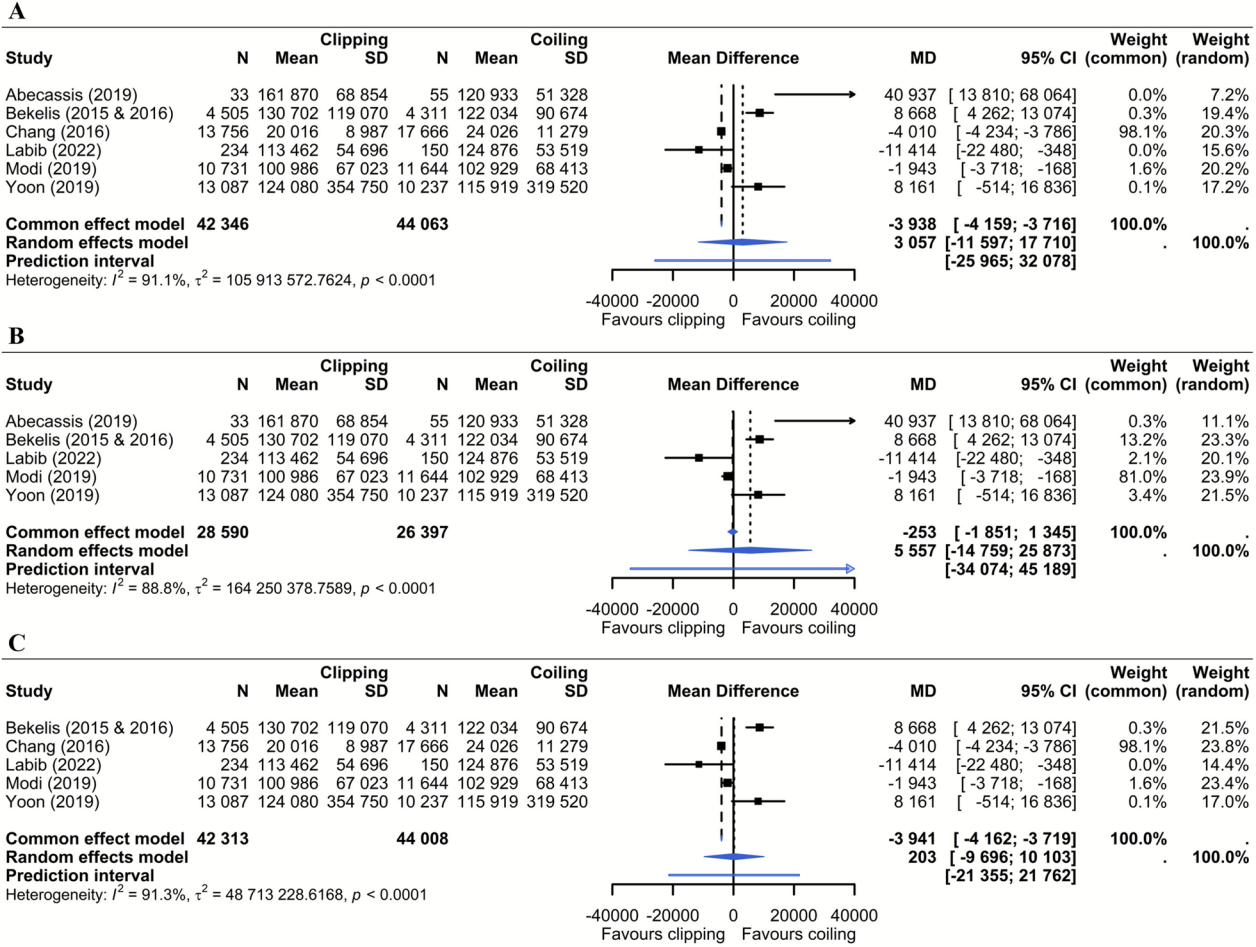
Forest plots. In-hospital costs of neurosurgical clipping versus endovascular coiling. Panel A presents the pooled analysis of all included studies. Bekelis et al. consists of two separate studies, one on clipping costs [23] and one on coiling costs [22], conducted by the same author with identical inclusion years and data source. This allowed for comparison and both studies were combined into a single forest plot entry. Panel B shows a sensitivity analysis excluding Chang et al. [25], the only study conducted outside the USA. Panel C shows a sensitivity analysis excluding Abecassis et al. [21], the study with the smallest sample size. Costs are expressed in 2024 USD. Negative mean difference values favor clipping and positive values favor coiling

Nine studies directly compared aneurysm clipping and coiling costs (Table 1) [21, 25, 27, 30, 32, 33, 38, 40, 48]. In addition, two studies by the same author examined clipping and coiling separately but were similar in year of inclusion, data source, and methodology, allowing comparison [22, 23]. Median costs were $100,041 (range $20,016–$459,579) for clipping and $113,075 (range $24,026–$448,803) for coiling. Of the 11 studies, 3 did not provide measures of variability (standard deviation, standard error, or 95% CI), and 1 reported hospital charges instead of actual costs. These studies were excluded, and the remaining seven studies were considered eligible for the meta-analysis. The results of the two separate studies [22, 23] were combined into a single study estimate for the analysis, resulting in six studies in the meta-analysis (Fig. 6). The pooled analysis of the random-effects model showed no significant difference in in-hospital costs between clipping and coiling (mean difference $3,057, 95% CI − $11,597 to $17,710). A sensitivity analysis excluding the only study in the analysis not from the United States [25] also showed no significant difference (mean difference $5,557, 95% CI − $14,759 to $25,873). However, the overall certainty of evidence (GRADE) is rated very low because of the imprecision, indirectness, and substantial heterogeneity in both the main and subgroup analyses (*I*^2^ = 91.1% and 88.8%, respectively; *P* < 0.0001), warranting cautious interpretation of the findings (Supplementary File 3).

## Discussion

In-hospital costs for aSAH are substantial but varied widely across the included studies. Reported costs ranged from $11,884 to $459,579, with a median of $68,711. When expressed as a percentage of GDP per capita, costs ranged from 35 to 639%. A pooled analysis using a random-effects model revealed no statistically significant difference in in-hospital costs between surgical clipping and endovascular coiling.

Although no previous systematic reviews have specifically assessed direct in-hospital costs for aSAH, our findings align with traumatic brain injury cost reviews, wherein costs per patient range from $2,744 to $517,713 (2024 USD) [50, 51]. In contrast, aSAH costs are generally higher than for other stroke types [51–53]. This likely relates to several major cost drivers found in the current review, such as the need for costly interventions, prolonged neurocritical care, and relatively high complication rates. Complications, including hydrocephalus, rebleeds, and infections, contribute substantially to total costs. In particular, we found that DCI is associated with a 25–30% increase in costs, consistent with previous findings [54].

The cost variability observed in this review, as well as in other health care cost reviews, can largely be attributed to considerable heterogeneity in economic evaluations, which complicates comparisons. Differences in cost components, methodological approaches (e.g., hospital charges vs. actual costs), and the adopted economic perspectives are major sources of this variability [11]. Moreover, differences in reimbursement structures and resource allocation models, such as fee for service, bundled payments or diagnosis-related group systems, contribute to the variability by influencing how hospital costs are reported.

Regional differences are particularly notable, with the United States reporting significantly higher costs than Europe, Asia, and other regions. This reflects broader systemic factors, such as the organization of health care delivery, the pricing and valuation of medical services, and the degree of central regulation within each health system [55]. Institutional characteristics, such as hospital level, case volume, treatment protocols, and operational efficiency, further contribute to cross-country variability in reported costs. In high-income settings, device and labor costs dominate expenditures, whereas in lower-income regions, lower device and labor costs, limited access to endovascular technologies, and lower treatment intensity result in lower overall in-hospital costs. Nevertheless, some studies from low-income and middle-income countries showed costs exceeding twice the national GDP per capita, raising concerns about the affordability and access to health care [56]. However, only 5 of the 30 included studies were conducted in low-income and middle-income countries, highlighting a geographic disparity that limits global applicability and generalizability.

Mortality rates were largely comparable between Europe and North America, even though North America reported substantially higher in-hospital costs. Both regions showed mortality within a similar range, indicating that higher spending does not necessarily translate into better outcomes within high-income regions. In contrast, studies that reported the lowest in-hospital costs showed distinctly higher mortality. This suggests that lower costs reflect underresourced health care environments in which limited intensive care unit (ICU) capacity, inadequate monitoring, staffing shortages, and equipment constraints restrict access to specialized neurocritical care, which may account for the higher mortality observed [56]. Because of infrequent reporting of functional outcomes, it remains uncertain whether increased in-hospital expenditures ultimately translate into improved long-term recovery or quality of life.

Several major cost drivers identified in this review represent potentially modifiable aspects of care. In many included studies, ICU and hospital stays were extended by routine observation for DCI, with patients often remaining for two to three weeks in the ICU or ward regardless of clinical stability. Individualized fast-track pathways that accelerate transfer from ICU to the ward or support early discharge home can tailor monitoring intensity to patient risk and may reduce resource use without compromising safety [57]. However, as DCI is a common trigger for unplanned readmission, premature discharge may shift costs rather than avoid them. Targeted ward or outpatient surveillance with clear thresholds for reassessment may allow earlier discharge while maintaining patient safety. Hydrocephalus can also prolong intensive care stay, as patients may remain admitted while awaiting improvement or decisions regarding cerebrospinal fluid diversion. Establishing clear criteria for the timing of interventions, including when to place a drain, when to discontinue cerebrospinal fluid drainage, and when to escalate to permanent diversion, may reduce unnecessary monitoring days and resource use [58]. A further opportunity for cost reduction relates to neuroradiological imaging. In the included studies, imaging accounted for up to 12% of total costs. Imaging frequency and follow-up imaging often reflect routine practice rather than clinical need, and repeated CT, CT perfusion, or angiography does not consistently change treatment decisions. Reducing unnecessary scans and limiting advanced imaging to situations in which results are expected to influence management may decrease avoidable expenditure [59]. Together, targeted improvements in monitoring intensity, hydrocephalus management, and imaging use offer opportunities to reduce costs while maintaining high-quality care.

The pooled analysis found no significant difference in total in-hospital costs between surgical clipping and endovascular coiling, although the certainty of this finding was rated very low. The apparent cost similarity likely reflects the higher device expenses of coiling being offset by its shorter hospital stays, whereas the lower procedural costs of clipping are counterbalanced by longer inpatient stays that increase resource use [60]. Variations in case mix, patient severity, complication burden, and methodologic approach (e.g., cost components included, calculation methods, and economic perspective) further influence the cost patterns observed between the included studies. Additionally, institutional factors, including treatment setting, case volume, practitioner expertise, and local procurement costs, significantly shape the overall cost balance. However, although acute costs appear similar, coiling may lead to higher long-term costs due to lower aneurysm occlusion rates and increased risks of rebleeding and retreatment [61, 62]. Conversely, advances in endovascular device technology may gradually shift the cost equilibrium over time through broader adoption and market competition, underscoring the need for ongoing economic monitoring. Ultimately, without robust long-term data, firm conclusions about the relative cost-effectiveness of these approaches remain premature.

The overall quality of the included studies was moderate, with considerable variation. Most reported general study characteristics adequately. However, essential components of economic evaluation, such as detailed cost breakdowns, a reference year, and appropriate valuation methods (e.g., bottom-up costing approach), were often missing or inconsistently applied. Checklists such as CHEERS have contributed to improving the quality of economic evaluations in health care by providing structured guidance, yet their use as scoring tools in systematic reviews presents challenges [12]. A recent review identified 18 different checklists used to assess the quality of economic evaluations, with many applied inappropriately through unsuitable selection, incorrect application, or misinterpretation of results [63, 64]. Furthermore, these checklists can be highly subjective and may lack generalizability, particularly when economic evaluation is not the primary study objective. This underscores the need for more rigorous, transparent, and standardized economic evaluation methods supported by clear guidelines to improve consistency and comparability across studies.

### Limitations

This systematic review has several limitations. The exclusion of studies published before 2013 may have affected the comprehensiveness of this review and limits the ability to assess long-term cost trends. However, advances in neurocritical care, microsurgical and endovascular treatment, and differences in hospital resource management might make older cost data less representative of current practice. Furthermore, only six studies were available for the clipping versus coiling meta-analysis, precluding reliable assessments of reporting bias (funnel plot and Egger’s test) [17, 65]. The very low certainty of evidence due to substantial heterogeneity and potential confounding from differences in case mix, patient severity, and complication burden further limits comparability, warranting a cautious interpretation of the meta-analysis results. Additionally, an important limitation is the underestimation of total costs due to the exclusion of indirect and intangible costs, especially productivity loss, which is substantial in aSAH, given its onset at a relatively young age and major impact on lives [4, 43].

Future research should aim to provide a more comprehensive picture of the total economic burden of aSAH by including long-term follow-up and indirect costs, such as those associated with postdischarge care, rehabilitation, and lost productivity. Additionally, future work should evaluate how reducing redundant monitoring and imaging could streamline care and should work toward standardized protocols for DCI detection and complication management. There is also a need for cost-effectiveness analyses directly comparing clipping and coiling in aSAH, particularly across varied health care systems and economic settings. Finally, the development of a universally accepted and validated checklist tailored to economic evaluations in neurovascular disease would strengthen methodological consistency and the quality of future reviews.

## Conclusion

In-hospital costs for aSAH management are substantial, with considerable variability between studies driven by differences in health care systems between countries, costing methodologies between studies and clinical practices. Although clipping and coiling show comparable short-term hospital costs, the certainty of this finding is low, and economic factors should not outweigh clinical or anatomical considerations when selecting a treatment strategy. The overall quality of economic evaluations remains inconsistent, highlighting the need for more standardized, transparent, and methodologically robust studies to better inform health care planning and resource allocation. Ultimately, improving transparency in economic reporting and streamlined treatment pathways that eliminate redundant resource use are essential to ensure efficient and sustainable care.

## Supplementary Information

Below is the link to the electronic supplementary material.

Supplementary file1 (DOCX 16 KB)

Supplementary file2 (DOCX 54 KB)

Supplementary file3 (DOCX 17 KB)
