# Supplementary material for: Systematic Review of Direct Hospital Costs Associated with Aneurysmal Subarachnoid Hemorrhage Management
Source: Neurocrit Care. 2026 Jan 15;44(2):680–99. doi: 10.1007/s12028-025-02439-2 (PMC13053336; doi:10.1007/s12028-025-02439-2)
Supplement: Supplementary file 1 — Supplementary file1 (DOCX 16 KB) [file 12028_2025_2439_MOESM1_ESM.docx]

**Pubmed**

("Economics"[MeSH] OR "Costs and Cost Analysis"[MeSH] OR "Costs and Cost Analys*"[ti] OR "Cost Analysis" [ti] OR "Cost Comparison*" [ti] OR "Cost of Illness"[MeSH] OR "Cost of Illness"[ti] OR "Cost Measure*"[ti] OR "cost"[ti] OR "costs"[ti] OR "economic*"[ti] OR "financ*"[ti] OR "money*"[ti] OR "charg*"[ti]) AND ("Subarachnoid hemorrhage"[MeSH] OR "SAH"[tiab] OR "aSAH"[tiab] OR "subarachnoid hemorrhage"[tiab] OR "subarachnoid haemorrhage"[tiab] OR "subarachnoid hemorrhages"[tiab] OR "subarachnoid haemorrhages"[tiab] OR "intracranial aneurysm"[MeSH] OR "intracranial aneurysms"[tiab] OR "intracranial aneurysm"[tiab] OR "aneurysmal hemorrhage*"[tiab] OR "aneurysmal haemorrhage*"[tiab])

531 results on 29-05-2024

Updated 29-05-2024 – 01-07-2025: 41 results

**Web of Science**

(TS=Economics OR TS="Costs and Cost Analysis" OR TI="Costs and Cost Analys*" OR TI="Cost Analysis" OR TI="Cost Comparison*" OR TS="Cost of Illness" OR TI="Cost of Illness" OR TI="Cost Measure*" OR TI=cost OR TI=costs OR TI=economic* OR TI=financ* OR TI=money* OR TI=charg* ) AND (TS="Subarachnoid hemorrhage" OR (TI=SAH OR AB=SAH) OR (TI=asas OR AB=asas) OR (TI="subarachnoid hemorrhage" OR AB="subarachnoid hemorrhage") OR (TI="subarachnoid haemorrhage" OR AB="subarachnoid haemorrhage") OR (TI="subarachnoid hemorrhages" OR AB="subarachnoid hemorrhages") OR (TI="subarachnoid haemorrhages" OR AB="subarachnoid haemorrhages") OR TS="intracranial aneurysm" OR (TI="intracranial aneurysms" OR AB="intracranial aneurysms") OR (TI="intracranial aneurysm" OR AB="intracranial aneurysm") OR (TI="aneurysmal hemorrhage*" OR AB="aneurysmal hemorrhage*") OR (TI="aneurysmal haemorrhage*" OR AB="aneurysmal haemorrhage*"))

407 results on 29-05-2024

Updated 29-05-2024 – 01-07-2025: 21 results

**Embase**

("Subarachnoid hemorrhage" or "SAH" or "aSAH" or "subarachnoid hemorrhage" or "subarachnoid haemorrhage" or "subarachnoid hemorrhages" or "subarachnoid haemorrhages" or "intracranial aneurysm" or "intracranial aneurysms" or "intracranial aneurysm" or "aneurysmal hemorrhage*" or "aneurysmal haemorrhage*").ti,ab. and (costs or cost or expense or affordability or "financial burden").ti.

276 results on 29-05-2024

Updated 29-05-2024 – 01-07-2025: 16 results

**Emcare**

(exp Economics/ OR exp "Costs and Cost Analysis"/ OR "Costs and Cost Analys*".ti. OR "Cost Analysis".ti. OR "Cost Comparison*".ti. OR exp "Cost of Illness"/ OR "Cost of Illness".ti. OR "Cost Measure*".ti. OR cost.ti. OR costs.ti. OR economic*.ti. OR financ*.ti. OR money*.ti. OR charg*.ti.) AND (exp "Subarachnoid hemorrhage"/ OR SAH.tw. OR aSAH.tw. OR "subarachnoid hemorrhage".tw. OR "subarachnoid haemorrhage".tw. OR "subarachnoid hemorrhages".tw. OR "subarachnoid haemorrhages".tw. OR exp "intracranial aneurysm"/ OR "intracranial aneurysms".tw. OR "intracranial aneurysm".tw. OR "aneurysmal hemorrhage*".tw. OR "aneurysmal haemorrhage*".tw.)

211 results on 29-05-2024

Updated 29-05-2024 – 01-07-2025: 0 results

**Cochrane**

([mh "Economics"] OR [mh "Costs and Cost Analysis"] OR ("Costs and Cost" NEXT Analys*):ti OR "Cost Analysis":ti OR ("Cost" NEXT Comparison*):ti OR [mh "Cost of Illness"] OR "Cost of Illness":ti OR ("Cost" NEXT Measure*):ti OR cost:ti OR costs:ti OR economic*:ti OR financ*:ti OR money*:ti OR charg*:ti) AND ([mh "Subarachnoid hemorrhage"] OR SAH:ti,ab OR aSAH:ti,ab OR "subarachnoid hemorrhage":ti,ab OR "subarachnoid haemorrhage":ti,ab OR "subarachnoid hemorrhages":ti,ab OR "subarachnoid haemorrhages":ti,ab OR [mh "intracranial aneurysm"] OR "intracranial aneurysms":ti,ab OR "intracranial aneurysm":ti,ab OR ("aneurysmal" NEXT hemorrhage*):ti,ab OR ("aneurysmal" NEXT haemorrhage*):ti,ab)

27 results on 29-05-2024

Updated 29-05-2024 – 01-07-2025: 0

**PsycInfo**

TX ( "Subarachnoid hemorrhage" OR "SAH" OR "aSAH" OR "subarachnoid hemorrhage" OR "subarachnoid haemorrhage" OR "subarachnoid hemorrhages" OR "subarachnoid haemorrhages" OR "intracranial aneurysm" OR "intracranial aneurysms" OR "intracranial aneurysm" OR "aneurysmal hemorrhage*" OR "aneurysmal haemorrhage*" ) AND TI ( costs or cost or expense or affordability or financial burden)

11 results on 29-05-2024

Updated 29-05-2024 – 01-07-2025: 0
