# Supplementary material for: Systematic Review of Direct Hospital Costs Associated with Aneurysmal Subarachnoid Hemorrhage Management
Source: Neurocrit Care. 2026 Jan 15;44(2):680–99. doi: 10.1007/s12028-025-02439-2 (PMC13053336; doi:10.1007/s12028-025-02439-2)
Supplement: Supplementary file 2 — Supplementary file2 (DOCX 54 KB) [file 12028_2025_2439_MOESM2_ESM.docx]

| **Supplementary Table 1.** Study Reporting Quality CHEERS 2022 | | | | | | | | | | | | | | | | | | | | | | | | | | | | | | | |
| --- | --- | --- | --- | --- | --- | --- | --- | --- | --- | --- | --- | --- | --- | --- | --- | --- | --- | --- | --- | --- | --- | --- | --- | --- | --- | --- | --- | --- | --- | --- | --- |
| **Author** | **1** | **2** | **3** | **4** | **5** | **6** | **7** | **8** | **9** | **10** | **11** | **12** | **13** | **14** | **15** | **16** | **17** | **18** | **19** | **20** | **21** | **22** | **23** | **24** | **25** | **26** | **27** | **28** | **Total Score** | **Maximum Score** | **Percentage** |
| Abdo | 2 | 2 | 2 | 0 | 2 | 2 | NA | 1 | 2 | NA | 2 | 2 | NA | 2 | 1 | NA | 2 | 2 | 2 | 1 | 0 | 2 | 2 | 1 | NA | 2 | 2 | 2 | 38 | 46 | **83** |
| Abecassis | 2 | 2 | 2 | 0 | 2 | 2 | 2 | 1 | 2 | 1 | 2 | 2 | 2 | 2 | 0 | NA | 2 | 1 | 0 | 2 | 0 | 2 | 2 | 1 | NA | 2 | 2 | 2 | 40 | 52 | **77** |
| Bekelis | 2 | 2 | 2 | 0 | 2 | 2 | NA | 2 | 2 | NA | 2 | 2 | NA | 2 | 2 | NA | 2 | 2 | 2 | 2 | 0 | 2 | 2 | 1 | NA | 2 | 0 | 2 | 39 | 46 | **85** |
| Bekelis | 2 | 2 | 2 | 0 | 2 | 2 | NA | 1 | 2 | NA | 2 | 2 | NA | 2 | 1 | NA | 2 | 2 | 2 | 2 | 0 | 2 | 2 | 1 | NA | 2 | 2 | 2 | 39 | 46 | **85** |
| Calciolari | 2 | 2 | 2 | 1 | 2 | 2 | NA | 2 | 2 | NA | 2 | 2 | NA | 2 | 2 | NA | 2 | 2 | 0 | 2 | 0 | 2 | 2 | 2 | NA | 2 | 2 | 2 | 41 | 46 | **89** |
| Chang | 2 | 2 | 1 | 0 | 1 | 2 | 1 | 1 | 2 | NA | 0 | 0 | 0 | 1 | 1 | NA | 1 | 0 | 0 | 0 | 0 | NA | 2 | NA | NA | 2 | 2 | 0 | 21 | 46 | **46** |
| Chen | 0 | 1 | 1 | 0 | 2 | 2 | 2 | 0 | 2 | NA | 1 | 2 | NA | 1 | 0 | NA | 2 | 1 | 0 | 1 | 0 | 2 | 2 | 1 | NA | 2 | 2 | 1 | 28 | 48 | **58** |
| Deutsch | 0 | 1 | 1 | 0 | 2 | 2 | 2 | 1 | 1 | NA | 1 | 1 | NA | 1 | 0 | NA | 2 | 2 | 0 | 1 | 0 | 2 | 2 | 1 | NA | 2 | 1 | 2 | 28 | 48 | **58** |
| Fernando | 2 | 2 | 2 | 2 | 2 | 2 | NA | 1 | 2 | NA | 1 | 2 | NA | 2 | 2 | NA | 1 | 2 | 0 | 0 | 0 | NA | 2 | NA | NA | 2 | 2 | 2 | 33 | 42 | **79** |
| Koester | 2 | 2 | 2 | 0 | 2 | 2 | 2 | 1 | 2 | NA | 2 | 2 | NA | 2 | 1 | NA | 2 | 1 | 0 | 1 | 0 | 2 | 2 | 1 | NA | 2 | 2 | 2 | 37 | 48 | **77** |
| Labib | 2 | 2 | 2 | 0 | 2 | 2 | 2 | 1 | 2 | NA | 1 | 2 | NA | 2 | 0 | NA | 2 | 1 | 0 | 1 | 0 | 2 | 2 | 1 | NA | 2 | 2 | 2 | 35 | 48 | **73** |
| Lee | 2 | 2 | 2 | 0 | 2 | 2 | NA | 2 | 2 | NA | 2 | 2 | NA | 2 | 0 | NA | 2 | 2 | 0 | 1 | 0 | 2 | 2 | 1 | NA | 1 | 2 | 0 | 33 | 46 | **72** |
| Modi | 2 | 2 | 2 | 0 | 2 | 2 | NA | 2 | 2 | NA | 0 | 1 | NA | 1 | 1 | NA | 2 | 2 | 0 | 1 | 0 | 1 | 2 | 1 | NA | 2 | 2 | 2 | 32 | 46 | **70** |
| Monsivais | 2 | 2 | 2 | 0 | 2 | 2 | 2 | 2 | 2 | NA | 1 | 2 | NA | 2 | 2 | NA | 1 | 2 | 0 | 0 | 0 | NA | 2 | NA | NA | 2 | 2 | 2 | 34 | 44 | **77** |
| Murata | 1 | 2 | 2 | 1 | 2 | 2 | NA | 2 | 2 | NA | 2 | 2 | NA | 2 | 1 | NA | 2 | 2 | 0 | 0 | 0 | NA | 2 | NA | NA | 2 | 2 | 0 | 31 | 42 | **74** |
| Ng | 2 | 2 | 2 | 1 | 2 | 2 | NA | 2 | 2 | NA | 1 | 2 | NA | 2 | 2 | NA | 1 | 2 | 1 | 1 | 0 | 2 | 2 | 1 | NA | 2 | 0 | 2 | 36 | 46 | **78** |
| Ojha | 0 | 1 | 1 | 0 | 2 | 2 | NA | 1 | 2 | NA | 1 | 2 | NA | 2 | 0 | NA | 1 | 1 | 1 | 1 | 0 | NA | 2 | NA | NA | 2 | 2 | 2 | 26 | 42 | **62** |
| Raj | 2 | 2 | 2 | 0 | 2 | 2 | NA | 1 | 2 | NA | 2 | 2 | 2 | 2 | 1 | NA | 2 | 1 | 0 | 2 | 0 | 2 | 2 | 2 | NA | 2 | 2 | 2 | 39 | 48 | **81** |
| Ramos | 2 | 2 | 2 | 0 | 2 | 2 | 2 | 1 | 2 | NA | 2 | 2 | NA | 2 | 1 | NA | 2 | 2 | 2 | 1 | 0 | 2 | 2 | 1 | NA | 2 | 0 | 2 | 38 | 48 | **79** |
| Rha | 2 | 2 | 2 | 0 | 2 | 2 | NA | 2 | 2 | NA | 1 | 2 | NA | 2 | 1 | NA | 0 | 2 | 0 | 0 | 0 | NA | 1 | NA | NA | 2 | 2 | 2 | 29 | 42 | **69** |
| Ridwan | 2 | 2 | 2 | 0 | 2 | 2 | 2 | 2 | 2 | NA | 2 | 2 | NA | 2 | 2 | NA | 1 | 0 | 0 | 1 | 0 | 0 | 2 | 1 | NA | 2 | 2 | 2 | 35 | 48 | **73** |
| Rumalla | 0 | 2 | 1 | 0 | 2 | 2 | NA | 1 | 2 | NA | 1 | 2 | NA | 2 | 0 | NA | 2 | 0 | 0 | 1 | 0 | 2 | 2 | 1 | NA | 2 | 2 | 2 | 29 | 46 | **63** |
| Safanelli | 2 | 2 | 2 | 1 | 2 | 2 | NA | 1 | 2 | NA | NA | NA | NA | 2 | 2 | NA | 1 | 2 | 0 | 0 | 0 | NA | 2 | NA | NA | 2 | 2 | 2 | 29 | 38 | **76** |
| Seule | 2 | 2 | 2 | 0 | 2 | 2 | 2 | 2 | 2 | NA | 2 | 2 | 2 | 2 | 2 | NA | 2 | 2 | 0 | 0 | 0 | NA | 2 | NA | NA | 2 | 0 | 2 | 36 | 46 | **78** |
| Stepanova | 1 | 2 | 2 | 0 | 2 | 2 | 2 | 0 | 1 | NA | 1 | 2 | NA | 1 | 1 | NA | 2 | 2 | 1 | 1 | 0 | 2 | 2 | 1 | NA | 2 | 2 | 2 | 34 | 48 | **71** |
| Timmers | 2 | 2 | 2 | 0 | 2 | 2 | 2 | 2 | 2 | NA | 2 | 2 | NA | 2 | 0 | NA | 2 | 1 | 0 | 1 | 0 | 2 | 2 | 1 | NA | 2 | 2 | 2 | 37 | 48 | **77** |
| Tong | 2 | 1 | 1 | 0 | 2 | 2 | 2 | 1 | 2 | NA | NA | NA | NA | 2 | 1 | NA | 2 | 2 | 0 | 1 | 0 | 2 | 2 | 1 | NA | 2 | 2 | 2 | 32 | 44 | **73** |
| Xu | 2 | 2 | 2 | 0 | 2 | 2 | NA | 1 | 1 | NA | 2 | 1 | NA | 0 | 2 | NA | 2 | 2 | 1 | 1 | 0 | 2 | 2 | 1 | NA | 2 | 2 | 2 | 34 | 46 | **74** |
| Yoon | 2 | 2 | 2 | 0 | 2 | 2 | 2 | 1 | 2 | NA | 1 | 1 | NA | 1 | 1 | NA | 2 | 1 | 2 | 1 | 0 | 1 | 2 | 1 | NA | 2 | 1 | 1 | 33 | 48 | **69** |
| Zhang | 2 | 2 | 2 | 1 | 2 | 2 | NA | 2 | 2 | NA | 2 | 2 | NA | 2 | 1 | NA | 2 | 2 | 2 | 2 | 0 | 2 | 2 | 2 | NA | 2 | 2 | 2 | 42 | 46 | **91** |

| **Consolidated Health Economic Evaluation Reporting Standards 2022** | |
| --- | --- |
| **Item** | **Guidance for Reporting** |
| 1. Title | Identify the study as an economic evaluation and specify the interventions being compared. |
| 2. Abstract | Provide a structured summary that highlights context, key methods, results and alternative analyses. |
| 3. Background and objectives | Give the context for the study, the study question and its practical relevance for decision making in policy or practice. |
| 4. Health economic analysis plan | Indicate whether a health economic analysis plan was developed and where available. |
| 5. Study population | Describe characteristics of the study population (such as age range, demographics, socioeconomic, or clinical characteristics). |
| 6. Setting and location | Provide relevant contextual information that may influence findings. |
| 7. Comparators | Describe the interventions or strategies being compared and why chosen. |
| 8. Perspective | State the perspective(s) adopted by the study and why chosen. |
| 9. Time horizon | State the time horizon for the study and why appropriate. |
| 10. Discount rate | Report the discount rate(s) and reason chosen. |
| 11. Selection of outcomes | Describe what outcomes were used as the measure(s) of benefit(s) and harm(s). |
| 12. Measurement of outcomes | Describe how outcomes used to capture benefit(s) and harm(s) were measured. |
| 13. Valuation of outcomes | Describe the population and methods used to measure and value outcomes. |
| 14. Measurement and valuation of costs | Describe how resources and costs were valued. |
| 15. Currency, price date, and conversion | Report the dates of the estimated resource quantities and unit costs, plus the currency and year of conversion. |
| 16. Rationale and description of model | If modelling is used, describe in detail and why used. Report if the model is publicly available and where it can be accessed. |
| 17. Analytics and assumptions | Describe any methods for analysing or statistically transforming data, any extrapolation methods, and approaches for validating any model used. |
| 18. Characterizing heterogeneity | Describe any methods used for estimating how the results of the study vary for sub-groups. |
| 19. Characterizing distributional effects | Describe how impacts are distributed across different individuals or adjustments made to reflect priority populations. |
| 20. Characterizing uncertainty | Describe methods to characterize any sources of uncertainty in the analysis. |
| 21. Approach to engagement with patients and others affected by the study | Describe any approaches to engage patients or service recipients, the general public, communities, or stakeholders (e.g., clinicians or payers) in the design of the study. |
| 22. Study parameters | Report all analytic inputs (e.g., values, ranges, references) including uncertainty or distributional assumptions. |
| 23. Summary of main results | Report the mean values for the main categories of costs and outcomes of interest and summarise them in the most appropriate overall measure. |
| 24. Effect of uncertainty | Describe how uncertainty about analytic judgments, inputs, or projections affect findings. Report the effect of choice of discount rate and time horizon, if applicable. |
| 25. Effect of engagement with patients and others affected by the study | Report on any difference patient/service recipient, general public, community, or stakeholder involvement made to the approach or findings of the study |
| 26. Study findings, limitations, generalizability, and current knowledge | Report key findings, limitations, ethical or equity considerations not captured, and how these could impact patients, policy, or practice. |
| 27. Source of funding | Describe how the study was funded and any role of the funder in the identification, design, conduct, and reporting of the analysis |
| 28. Conflicts of interest | Report authors conflicts of interest according to journal or International Committee of Medical Journal Editors requirements. |

| **Supplementary Table 2.** Study Scoring JBI | | | | | | | | | | | | | | |
| --- | --- | --- | --- | --- | --- | --- | --- | --- | --- | --- | --- | --- | --- | --- |
| **Author** | **1** | **2** | **3** | **4** | **5** | **6** | **7** | **8** | **9** | **10** | **11** | **Score** | **Maximum**  **Score** | **Percentage** |
| Abdo | 2 | NA | 2 | NA | 2 | 2 | 1 | NA | 0 | 1 | 2 | 12 | 16 | 75 |
| Abecassis | 2 | 2 | 2 | 2 | 2 | 1 | 0 | 2 | 1 | 2 | 2 | 18 | 22 | 82 |
| Bekelis | 2 | NA | 2 | NA | 1 | 1 | 2 | NA | 1 | 2 | 2 | 13 | 16 | 81 |
| Bekelis | 2 | NA | 2 | NA | 1 | 1 | 2 | NA | 1 | 2 | 2 | 13 | 16 | 81 |
| Calciolari | 2 | 2 | 2 | 2 | 2 | 2 | 2 | 2 | 2 | 2 | 2 | 22 | 22 | 100 |
| Chang | 2 | 1 | 1 | 1 | 1 | 1 | 0 | 0 | 0 | 2 | 2 | 11 | 22 | 50 |
| Chen | 2 | 2 | 0 | 2 | 0 | 0 | 0 | 1 | 0 | 1 | 1 | 9 | 22 | 41 |
| Deutsch | 2 | 2 | 1 | 2 | 1 | 0 | 0 | 2 | 1 | 2 | 2 | 15 | 22 | 68 |
| Fernando | 2 | NA | 2 | NA | 2 | 2 | 2 | NA | 1 | 2 | 2 | 15 | 16 | 94 |
| Koester | 2 | 2 | 2 | NA | 2 | 1 | 0 | 1 | 0 | 2 | 2 | 14 | 20 | 70 |
| Labib | 2 | 2 | 2 | 2 | 2 | 2 | 1 | 2 | 1 | 2 | 2 | 20 | 22 | 91 |
| Lee | 2 | NA | 2 | NA | 1 | 1 | 0 | NA | 1 | 1 | 2 | 10 | 16 | 63 |
| Modi | 2 | NA | 2 | NA | 1 | 1 | 2 | NA | 1 | 1 | 2 | 12 | 16 | 75 |
| Monsivais | 2 | 1 | 2 | 0 | 2 | 2 | 1 | 1 | 0 | 2 | 2 | 15 | 22 | 68 |
| Murata | 2 | NA | 2 | NA | 1 | 1 | 2 | NA | 1 | 2 | 2 | 13 | 16 | 81 |
| Ng | 2 | 2 | 2 | NA | 2 | 2 | 2 | NA | 1 | 2 | 2 | 17 | 18 | 94 |
| Ojha | 2 | NA | 2 | NA | 2 | 1 | 0 | NA | 1 | 2 | 2 | 12 | 16 | 75 |
| Raj | 2 | NA | 2 | NA | 2 | 1 | 2 | NA | 2 | 2 | 2 | 15 | 16 | 94 |
| Ramos | 2 | 2 | 0 | NA | 1 | 1 | 2 | NA | 0 | 1 | 1 | 10 | 18 | 56 |
| Rha | 2 | NA | 2 | NA | 2 | 1 | 2 | NA | 1 | 2 | 2 | 14 | 16 | 88 |
| Ridwan | 2 | NA | 1 | NA | 1 | 1 | 1 | NA | 1 | 1 | 2 | 10 | 16 | 63 |
| Rumalla | 2 | NA | 2 | NA | 1 | 1 | 0 | NA | 0 | 1 | 2 | 9 | 16 | 56 |
| Safanelli | 2 | 1 | 2 | NA | 2 | 2 | 2 | NA | 1 | 2 | 2 | 16 | 18 | 89 |
| Seule | 2 | 2 | 2 | NA | 1 | 2 | 2 | NA | 1 | 2 | 2 | 16 | 18 | 89 |
| Stepanova | 2 | NA | 2 | NA | 1 | 1 | 1 | NA | 1 | 2 | 2 | 12 | 16 | 75 |
| Timmers | 2 | 2 | 2 | NA | 1 | 1 | 0 | NA | 1 | 2 | 2 | 13 | 18 | 72 |
| Tong | 2 | NA | 2 | NA | 1 | 1 | 2 | NA | 1 | 2 | 2 | 13 | 16 | 81 |
| Xu | 2 | NA | 2 | NA | 1 | 0 | 1 | NA | 1 | 2 | 2 | 11 | 16 | 69 |
| Yoon | 2 | NA | 2 | NA | 1 | 1 | 2 | NA | 1 | 1 | 1 | 11 | 16 | 69 |
| Zhang | 2 | 2 | 2 | NA | 1 | 1 | 2 | NA | 2 | 2 | 2 | 16 | 18 | 89 |

| **Joanna Briggs Institute Critical Appraisal Checklist for Economic Evaluations** |
| --- |
| 1. Is there a well-defined question? |
| 1. Is there comprehensive description of alternatives? |
| 1. Are all important and relevant costs and outcomes for each alternative identified? |
| 1. Has clinical effectiveness been established? |
| 1. Are costs and outcomes measured accurately? |
| 1. Are costs and outcomes valued credibly? |
| 1. Are costs and outcomes adjusted for differential timing? |
| 1. Is there an incremental analysis of costs and consequences? |
| 1. Were sensitivity analyses conducted to investigate uncertainty in estimates of cost or consequences? |
| 1. Do study results include all issues of concern to users? |
| 1. Are the results generalizable to the setting of interest in the review? |
