# Supplementary material for: Systematic Review of Direct Hospital Costs Associated with Aneurysmal Subarachnoid Hemorrhage Management
Source: Neurocrit Care. 2026 Jan 15;44(2):680–99. doi: 10.1007/s12028-025-02439-2 (PMC13053336; doi:10.1007/s12028-025-02439-2)
Supplement: Supplementary file 3 — Supplementary file3 (DOCX 17 KB) [file 12028_2025_2439_MOESM3_ESM.docx]

| **Supplementary Table 3.** Certainty of evidence (GRADE) for clipping versus coiling | | | | |
| --- | --- | --- | --- | --- |
| **Outcome** | **Studies** | **Participants** | **Results** | **Certainty of evidence (GRADE)** |
| Cost difference clipping vs coiling | 6 | Clipping: 42,346  Coiling: 44,036 | Mean difference: $3057  95CI: -$11,597 – $17,710  *I^2^*= 91.1% | ⊕⊖⊖⊖  **Very low**^a,b,c,d,e^ |
| Explanations:  ^a^ Most included studies were observational and retrospective, certainty therefore started at “low” according to GRADE. Methodological quality (CHEERS, JBI) was overall moderate, with large variation, leading to downgrading for risk of bias.  ^b^ There was no apparent reason to suspect publication bias, as cost and length-of-stay data are routinely collected and reported regardless of study findings, therefore, no downgrade was applied.  ^c^ There was large variation in reported costs across studies, with substantial methodological and statistical heterogeneity (I² = 91.1%) also in the sensitivity analyses, leading to downgrading for inconsistency.  ^d^ The pooled estimate had a wide confidence interval crossing 0, leading to downgrading for imprecision.  ^e^ Downgrading was applied for indirectness because the included studies were conducted in diverse health systems using different costing methods and patient selection criteria, which may limit the applicability of the observed cost differences between clipping and coiling to other settings. | | | | |
